# The role of miR-16 and miR-34a family in the regulation of cancers: A review

**DOI:** 10.1016/j.heliyon.2025.e42733

**Published:** 2025-02-17

**Authors:** Zahra Sadeghi, Mehrnoush Malekzadeh, Mohammadreza Sharifi, Batool Hashemibeni

**Affiliations:** a Department of Anatomical Sciences and Reproductive Biology, School of Medicine, Isfahan University of Medical Sciences, Isfahan, Iran; b Department of Genetics and Molecular Biology, School of Medicine, Isfahan University of Medical Sciences, I.R, Isfahan, Iran

**Keywords:** Cancer, microRNAs, miR16-5p, miR- 34

## Abstract

microRNAs (miRNAs), regulatory non-coding RNAs, can change translation, and decrease protein expression. miR-16 and miR-34a families are among the most abundant tumor suppressors and highly conserved microRNAs recognized. They have vital regulatory roles in health and disease. Their regulatory functions include biological processes such as improvement, differentiation, cell death, survival, and cell metabolism. The use of miR-16 and miR-34a families as biomarkers for cancer treatment is likely to improve patients with cancer. In this review, we update on recent advances in understanding the mechanism of miR-16 and miR-34 families function in cancer. Knowing about these mechanisms is effective for improving drugs and treatment methods. We also evaluated the reviewed studies and by introducing their weaknesses, we made suggestions for improving future research.

## 1 Introduction

Since the discovery of regulatory and small non-coding RNAs called microRNAs (miRNAs) less than 35 years ago, the importance of obtaining information about their role in the control of the various biological activities in cells, such as growth, proliferation, survival, cell death, migration, differentiation, and invasion has increased. miRNAs contain 19-25 nucleotides [1,2]. Specifically, miRNAs are divided into oncogenes (oncomiRs) and tumor suppressors. OncomiRs act as tumor promoters by inhibiting tumor suppressor genes; and conversely, tumor suppressor miRNAs inhibit oncogenes, thus inhibiting tumor growth [3]. Therefore, it is said that miRNAs are key regulators in controlling cancer progression, invasion, and metastasis [4]. Thus when the expression of miRNAs disrupts, it can affect the cancer cell survival and the process of cancer treatment [5]. In this situation, it is vital to try to maintain the expression of tumor suppressor miRNAs. Also, nanoparticles can be used to deliver these miRNAs to tumoral cells. MiR-206 conjugated gold nanoparticle-based, targets therapy in breast cancer cells by blocking the G1/S transition by targeting cyclin D2 (CCND2) [6].

Among the tumor suppressor miRNAs, miR-16 and miR-34 families are prominent in cancer. Since Research about the expression of miRNAs in tumors and their metastases provides a promising way for cancer treatment, so in this review we summarized the mechanism of miR-16 and miR-34a family's function and their signaling pathway, which are tumor suppressors in various cancers. These findings can contribute to research related to cancer treatment.

## 2 The miR-16 family

**Fig. 1:**
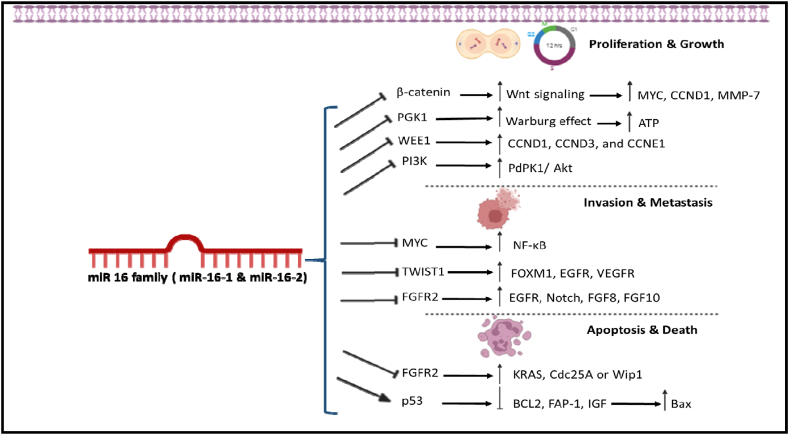
miR-16 family (miR-16-1 and miR-16-2) can modulate signaling pathway in tumoral cell. Upregulation the member of miR-16 family leads to the inhibition of invasion, metastasis, and cell growth as well as stimulation of apoptosis in cancer cells.

The miR-16 family (miR-16-1, miR-16-2, miR-16-5p) plays an inhibitor role in carcinogenesis (Fig. 1). miR-16 family is a biomarker for cancer diagnosis which affects various cell signaling pathways involved in tumor growth, proliferation, and invasion, although their expression level is different in cancers [7]. In colorectal cancer (CRC) cell lines SW480, HT-29 and Caco2, miR-16 inhibits tumor proliferation and metastasis of tumor cells by restoring Sensitivity to Tyrosine Kinase inhibitors, and targeting the transcription of 3′ untranslated regions (3′ UTRs) of KRAS and inhibiting KRAS's expression. Inhibiting KRAS is known to deactivate signal transduction pathways that stimulate tumorigenesis and resistance to therapies [8]. Ding et al. [9] explained that in cervical cancer (CC) which is a significant cancer in women of the world after breast cancer (BC) down-express of miR-16 happens. They showed that increased expression of miR-16 suppresses the cell cycle progression, proliferation of CC HCC94 cells with decreases the expression level of KRAS protein and increased apoptosis too. They also showed that miR-16 leads to apoptosis by targeting Cdc25A or Wip1 and modulating H2AX expression that induces autophagy.

BC, the first common cancer among women, leads to annual death. Upregulation of miR16 in BC suppresses the expression of genes related to epithelial-mesenchymal transition (EMT), such as transforming growth factor beta (TGF-β). During EMT, epithelial cells missed cell polarity and junctions and exchanged signaling pathways of cell shape and gene expression; thus increasing the motility of cells and creating an invasive phenotype with high expression of MYC. miR-16 inhibit MYC expression by suppressing of nuclear factor kappa-light-chain-enhancer of activated B cells (NF-κB) that leads to invasion and metastasis in BC [10].

In oral squamous cell carcinoma [11], ovarian cancer cells [12], and chordoma [13], miR-16 suppressed β-catenin proteins that have roles in Wnt signaling. β-catenin activated Wnt signaling and promotes the expression of its target genes, such as MYC, CCND1, and MMP-7 directly regulate the proliferation and migration [14]. CCND1 have a role in the G1/S phase of the cell cycle and promotes G1 to S phase and causes proliferation [15]. Briefly, miR-16 through some kinds of signaling molecular decelerates the progression of cancer. In the following, the detailed molecular mechanism of each member of the miR-16 family is discussed separately.

### 2.1 miR-16-1

miR-16-1, a member of the miR-16 family, located on the chromosome 13q (Table 1). miR-16-1 has a role in suppressing tumors in various malignancies such as chronic lymphocytic leukemia (CLL) by modulating molecules including B-cell lymphoma 2 (BCL2), Wnt, CCND, etc. In eukaryotic cells, BCL2 inhibits the death of cells and promotes cell survival too. Overexpression of the BCL2 gene has been reported in 65–70 % of CLL cells, and miR-16-1 by targeting BCL2 induces death in the leukemia cancer model. Reduction of BCL2 protein level by miR-16-1 leads to activation of BAX and BAK that bury themselves in the mitochondrial outer membrane, release cytochrome *c* from mitochondria, and shed light on the initiation of the apoptosis process [16].

**Table 1:** Antitumor effects of miR-16-1 and miR-16-2.

miR-16-1				
First author	year	Cancer	Results	Ref.
Ghaffari	2021	leukemia	targets BCL2, activates of BAX and BAK, releases cytochrome *c* from mitochondria, and increases apoptosis process	[16]
Cheng	2019	prostate cancer (PCa)	targets Wnt3a, encoding CCND1 and BCL2, which are involved in several tumorigenesis features, including cell proliferation, survival, and invasion	[17]
Braga	2022	lymphoma	Through the p53 protein activation mechanism increases tumor cell death. p53 directly deactivated the Bcl-2 protein, activated the Bax, and induced the release of cytochrome *c* and caspase-3 thus forming apoptotic bodies	[18]
Sargolzaei	2020	–	induced p53 and directly suppresses anti-apoptotic factors including FAP-1, Bcl2, and IGF, and induces cell death	[19]
Lu	2019	Brest cancer (BC)	by directly targeting the 3′ UTRs, suppresses BC cell metastasis and growth, and inhibits the phosphoglycerate kinase1 (PGK1) signaling pathway, a vital part in the glycolytic pathway that catalysis transformation of 1, 3-BPG and ADP into 3-phosphoglycerate (3-PG) and ATP, of the Warburg effect	[20]
Ye	2020	BC	reduces the transcription factor Twist-related protein 1 (TWIST1), which is activated by forkhead box protein M1 (FOXM1), epidermal growth factor (EGFR), and vascular endothelial growth factor receptor (VEGFR), to prevent the migration and invasion of BC cells	[21]

**miR-16**–**2**				
**First author**	**year**	**Cancer**	**Results**	**Ref.**

Ware	2022	bladder cancer	leads to the downregulation of CCND2 and cell cycle progression and so has inhibitory effects in malignant tumors	[23]
Quemener	2022	uveal melanoma (UM)	down-regulate cell cycle regulators such as WEE1 (a nuclear member of the protein kinases family), CCND3, and CCND1	[24]
Han Toraih	2019 2021	B-cell lymphomagenesis	inhibits the PdPK1/protein kinase B (Akt) signaling pathway, which promotes proliferation and growth in response to PI3K (phosphatidylinositol 3-kinase), and it may decrease cell proliferation and migration, and increase death through suppression of PDPK1	[26,27]
Maximov	2019	Osteosarcoma (OS)	targeting the FGFR2 and decrease angiogenesis, cell migration, and neural outgrowth, and promote cancer metastasis	[28]

Recently, Cheng et al. [17] showed that the miR-16-1 targets Wnt3a, encoding CCND1 and BCL2, which are involved in several prostate cancer (PCa) tumorigenesis features, including cell proliferation, survival, and invasion. It has been shown that miR-16-1 downregulation leads to prostate hyperplasia associated with the upregulation of CCND1 and Wnt3a, thus downregulation of this miRNA can lead to PCa development. Another signaling pathway for apoptosis that is directed by miR-16 is p53 protein activation, which is the tumor-suppressor feature of miRNAs. In research about lymphoma, seen that the expression of miR-16-1 through the p53 protein activation mechanism increases tumor cell death. p53 directly deactivated the Bcl-2 protein, activated the Bax, and induced the release of cytochrome *c* and caspase-3 thus forming apoptotic bodies [18]. In this regard, Sargolzaei et al. [19] confirmed miR-16-1 induced p53 and directly suppresses anti-apoptotic factors including FAP-1, Bcl2, and IGF, and induces cell death.

In addition to inducing apoptosis, miR-16-1 has a key role in reducing the proliferation and invasion of tumor cells. For example. miR-16-1-3p by directly targeting the 3′ UTRs, suppresses BC cell metastasis and growth, and inhibits the phosphoglycerate kinase1 (PGK1) signaling pathway, a vital part in the glycolytic pathway that catalysis transformation of 1, 3-BPG and ADP into 3-phosphoglycerate (3-PG) and ATP, of the Warburg effect. Warburg effect, known as aerobic glycolysis, is a phenomenon that favors glycolysis for energy, mainly in tumor cells; even when oxygen is sufficient [20]. miR-16-1-3p also inhibits tumor glycolysis in human BC by suppressing glucose uptake and lactate production. Therefore miR-16-1-3p regulates the PGK1-mediated Warburg effect and is essential in BC cell invasion, metastasis, migration, and proliferation. miR-16-1-3p acts as a tumor suppressor and reduces the transcription factor Twist-related protein 1 (TWIST1), which is activated by forkhead box protein M1 (FOXM1), epidermal growth factor (EGFR), and vascular endothelial growth factor receptor (VEGFR), to prevent the migration and invasion of BC cells [21].

### 2.2 miR-16-2

miR-16-2 is another member of the miR-16 family that is located on chromosome 3 (Table 1). Studies showed a decrease in the expression of miR-16-2-3p in patients with cancer such as thyroid cancer (TC) which is a malignant tumor of the endocrine system, thus the circulating level of miR-16-2 is a critical factor for the diagnosis of disease [22]. miR-16-2 leads to the downregulation of CCND2 and cell cycle progression and so has inhibitory effects in malignant tumors such as bladder cancer. In this study used extensive bioinformatics resources and accurate analysis of 304 males and 108 females [23]. Also, Quemener et al. [24] investigated the suppressor activity of miR-16-2 in the 1MP41 cell line of uveal melanoma (UM), a primary intraocular tumor that is a G protein-coupled receptor origin disease. Although treatment methods are not effective in curing UM metastasis they showed miR-16-2 has been observed to down-regulate cell cycle regulators such as WEE1 (a nuclear member of the protein kinases family), CCND3, and CCND1 in UM. miR-16-1 and miR-16-2 have the same sequence and exert similar biological effects and target CCND1, CCND3, and CCNE1 and affect cancer proliferation and survival. Also, in another cancer including B-cell lymphomas observation suggested a tumor suppressor role for the miR16-2. Deletion of miR-16-2 has an essential function in promoting mature B-cell lymphomagenesis [25].

3-phosphoinositide-dependent protein kinase-1 (PdPK1) of the AGc serine/threonine kinase family is necessary for cell proliferation, and invasion. miR-16-2-3p inhibits the PdPK1/protein kinase B (Akt) signaling pathway, which promotes proliferation and growth in response to PI3K (phosphatidylinositol 3-kinase), and it may decrease cell proliferation and migration, and increase death through suppression of PDPK1 [26,27]. Another signaling molecule that has a key role in cancer progression is fibroblast growth factor receptor 2 (FGFR2). miR-16-1-3p and miR-16-2-3p, targeting the FGFR2 and decrease angiogenesis, cell migration, and neural outgrowth, and promote cancer metastasis in Osteosarcoma (OS) and thus their upregulation sensitizes the cells to radiotherapy [28].

### 2.3 miR-16-5p

**Fig. 2:**
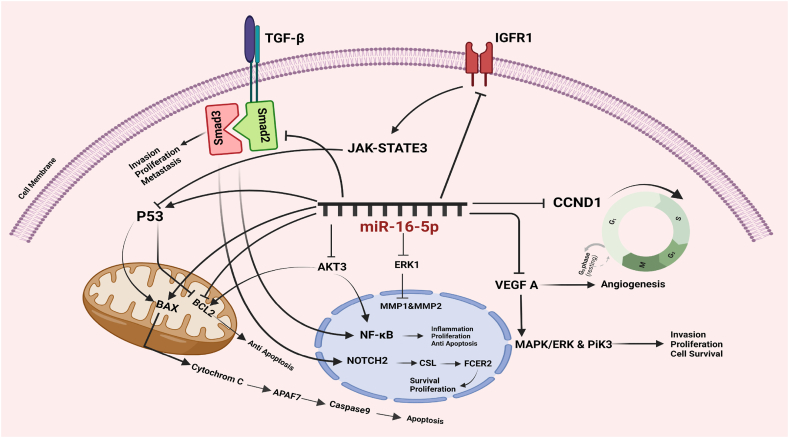
Mechanism of action of miR-16-5p in a cancer cell. The expression of miR-16-5p leads to the inhibition of invasion, metastasis, and proliferation well as stimulation of cell death in cancer cells.

miR-16-5p is another member of the miR-16 family known as a tumor suppressor (Fig. 2). miR-16-5p has low expression in most human cancers, including BC tissues [29]. While miR‐16‐5p was poorly expressed in cancer cells it is necessary to try to maintain its expression by affecting the molecular mechanisms of its function to slow down the cancer process.

#### 2.3.1 Cell proliferation and growth

It has been found that increased expression of TGF-β/anti-decapentaplegic 3 (Smad3) in cancers leads to decreased expression of miR-16-5p [30,31]. Ovarian cancer is asymptom in the early stages, and abdominal metastases are shown in more than 50 % of patients. miR-16-5p inhibits ovulation by inhibiting TGFβ1, which has a role in follicle development. Also, increased expression of miR-16-5p in ovarian cancer cells through interaction with target genes such as CCNE1, CHEK1, and VEGFA can decrease the proliferation [32]. miR-16-5p also by modulate cyclin and target CCND1 in PCa, cause cell cycle arrest in G0/G1 phase [33]. it increases the radio sensitivity in PCa via modulating the CCND1/E1-pRb-E2F1 signaling pathway [34]. miR-16-5p significantly inhibits tumorigenesis by reducing the expression of CCND1 [35]. miR-16-5p in glioblastoma cells may reduce glioma growth by down-regulating the cell cycle progression proteins (CDK6, CCND3, CCNE1, CDC25A) [36].

In human chondrosarcoma, cells via the PI3K/Akt signaling pathway, can promote tumor angiogenesis and suppress miR-16-5p expression. This pathway is under the affected of adipokine that it is related to various cancers, obesity, and inflammation and induces VEGF-A production; therefore, adipokine resistin through the reduction of expression of miR-16-5p and enhances VEGF-A and tumor angiogenesis [37]. Increased expression of miR-16-5p prevents cell proliferation by targeting VEGFA and ANLN genes. ANLN affects the proliferation of cells and controls the cell cycle. Eliminating ANLN can induce cell cycle arrest and inhibit the proliferation of cells in carcinoma. It also inhibits the invasion and metastasis of MCF7 and MDA-MB-231 cell lines. miR-16-5p in BC cells can decrease the expression of ANLN and suppressed metastasis, proliferation, and invasion. These changes happen via cell arrest in the G2/M phase of the cell cycle and increase cell death [38].

#### 2.3.2 Cell invasion and metastasis

miR-16-5p inhibits lung cancer proliferation by targeting Smad3 [39]. Expression of miR-16-5p in Chordoma, a rare tumor of mesenchymal tissue, reduces. In Chordoma, cell invasion and migration are suppressed and associated with upregulated E-cadherin expression and reduced N-cadherin expression. Also, Smad3 is a potential target of miR-16-5p. In Chordoma tissues, Smad3 is highly expressed and is related to tumor invasion [13].

#### 2.3.3 Cell apoptosis

In BC, decreased expression of miR-16-5p in addition to stimulating tumor cell proliferation, migration, and DNA synthesis in the cell cycle can inhibit apoptosis. AKT3's high expression in tumor cells leads to NF-κβ activating transcription factor polymerase 2, increasing inflammation, and modulating the expression of genes. So miR-16-5p prevents the progression of BC by inhibiting the NF-κβ and AKT3 pathways [40]. On the other hand, overexpression of miR-16-5p suppresses the inflammation response and inhibits the effect on PI3K kinase, a main component in the NF-κβ pathway [41,42]. miR-16-5p can target directly AKT3 in PCa. Overexpression of miR-16-5p and downregulation of AKT3 suppresses cell viability of PCa, regulates cell cycle distribution, and induces apoptosis [43]. The PIK3/Akt signaling pathway is significant in survival cells. miR-16-5p by PTEN, lipid, and protein with phosphatase activity, activation leads to inhibition of the PI3K/Akt pathway. It has been confirmed that PTEN blocks the PI3K/Akt signaling pathway through its phosphatase activity thus inhibiting cancer [20,44].

Also, miR-16-5p induces autophagy by targeting beclin 1, which is an important mediator of apoptosis and autophagy. The BCL2 homology region of beclin 1 interacts with BCL2/Bcl-xl proteins [45]. Another pathway that miR-16-5p modulates apoptosis is targeting BCL2. The level of BCL2 is increased with the simultaneous reduction of miR-16-5p, which inhibits apoptosis. Disruption of BCL2 activity inhibits proliferation, promotes death, and accelerates bone formation in OS [46].

CARM1 is another target gene of miR-16-5p that is related to apoptosis. The expression of CARM1 in healthy tissues is significantly lower than in cancer tissues. It promotes proliferation and metastasis in cancer cells and regulates the expression of p53 and NF-κβ. By targeting CARM1, miR-16-5p increases the sensitivity to radiotherapy in patients with CC [47].

miR-16-5p can also suppress the growth of CRC through downregulation of Integrin alpha 2 (TGA2), and stimulate apoptosis of CRC cells. Overexpression of miR-16-5p through inhibition of ITGA2 suppresses CRC progression, resulting in enhanced apoptosis of CRC cells. This research explained that the anti-ITGA2 antibody induces apoptosis by increasing the expression of RhoA-p38 MAPK, Bim, Apaf-1, and Caspase-9. However, the expressions of Ras and Bax/Bcl-2 were not affected [48].

## 3 The miR-34 family

The miR-34 family has two different transcription units. miR-34 family involved miR-34a, miR-34b, and miR-34c that located respectively on chromosome 22p and chromosome 11q. The expression of miR-34a is higher than other membranes. The highest level of expression in miR-34a and miR-34b/c is respectively in the brain, and lung [29]. Different mechanisms regulate the miR-34 expression. miR-34a and miR-34b/c have different antitumor potentials in carcinoma cells [49].

**Fig. 3:**
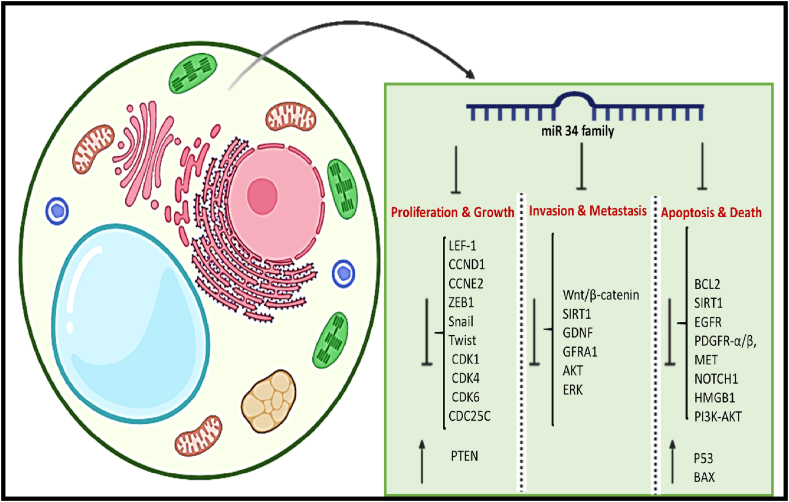
miR-34 family has suppressor effects on cancer invasion, proliferation, and metastasis by regulating molecular pathway. It also increase apoptosis in cancer cell.

Among the antitumor properties of miR-34 (Fig. 3), we can mention the increase in the process of cell death. For example in small-cell lung cancer (SCLC), expression of the miR-34 family is lower than in healthy tissues. Maintaining the expression of miR-34a can increase cell death and cell cycle arrest in G0/G1 and bind to Dual Specificity Phosphatase 1 (DUSP1), and it is a new role in the suppression of tumors in pathogenesis. it plays a key role by targeting c-Jun N-terminal kinases (JNK), autophagy suppressor by blocking FOXO1-mediated transcriptional activation of Bnip3, and preventing the release of Beclin-1 from Bcl-xL, induced apoptosis [50].

The miR-34 family can modulate EMT. Through EMT, most tumor cells can acquire tumor metastasis and invasion ability, leading to poor prognosis and patient death. miR-34c binds to the 3′UTR of Notch4 in BC, inhibits the ability of cell migration and the expression of mesenchymal markers, and enhances the expression of epithelial markers such as E-cadherin. At the initiation of EMT, a transcription factor SNAIL is produced. The feedback controls the EMT and is a direct target of the miR-34 family [29,51]. Another gen that is relative to EMT is Lymphatic enhancer factor 1 (LEF-1) which is related to cell proliferation and metastases. The expression of LEF-1 decreased by miR-34a in Pca [52]. In addition, the miR-34 family controls the TGF-β/Smad pathway and so inhibits EMT. miR-34 reduces the expression of TGF-βR1, p53, and phosphorylation of Smad3. Therefore, the migration of PCa cells is reduced, and invasion is weakened [29]. In the following, the detailed molecular mechanism of each member of the miR-34 family is discussed separately.

### 3.1 miR-34a

miR-34a has a role in the suppression of tumors (Table 2). Regulation of miR-34a in many cancers decreases but the point to think about here is whether its expression increases in thyroid carcinomas and their cell lines [53]. The findings showed that miR-34a has antitumor effects via three mechanisms: 1) Induces G0/G1 arrest and decreases cell proliferation. 2) Down-regulates EMT and suppresses cell motility. 3) Inhibits cancer cell autophagy and induces apoptosis. These functions lead to cancer cell chemosensitivity [54].

**Table 2:** Antitumor effects of miR-34a.

First author	year	Cancer	Results	Ref.
Chen	2017	TC	Active the PI3K/Akt/Bad signaling pathway and decreased Akt, have pro-growth and anti-apoptotic effects of miR-34a	[60]
Zhang	2017	CRC	miR-34a by binding to the 3′-UTR of and Jagged1 Notch1, inhibit the metastase	[61]
Chen	2017	TC	miR-34a regulate FDG by targeting GLUT1 and prevents metastasis	[60]
Saber Imani	2018	BC	miR-34a leads to p53 activation and apoptosis induction	[62]
Chen	2019	Urinary System Cancer	miR-34a reduces drug resistance through the inhibition of CD44	[63]
L Wei	2019	nasopharyngeal carcinoma	Decreased miR-34a expression in nasopharyngeal carcinoma leads to increased Ki67 expression level and tumor metastasis.	[64]
K Hasakova	2019	CRC	miR-34a leads to a decrease in the expression of the clock gene PER2, e IL-6 and STAT3 and has a role in the prevention of tumor metastasis.	[65]
Zhang	2019	various cancers	IL-6R is an oncogenic transcription factor that mediating the activation of STAT3. STAT3 binds to the site of miR-34a and suppresses the expression of miR-34a	[29]
Kalfert	2020	TC	miR-34a inhibition of SIRT1, increased p53 acetylation, activation apoptosis	[55]
Luo	2020	PCa	miR-34a target Notch-1 and inhibit cancer development	[66]
Wu	2021	HNSCC	miR-34a suppresses tumors by directly targeting BCL2, PIK3R2, c-Myc, SIRT1, VAMP2, MYH9, KLRK1, SDK4-6, Notch1, and CCR1	[67]
Li	2021	cancer stem cells	miR-34a target NOTCH, MYC, BCL2, and CD44 and suppress proliferation and invasion of cance cell	[45]
Lin	2022	PCa	miR-34a targeted LEF1 and EphA2 and inhibited proliferation and increased apoptosis	[68]
Shaban	2022	BC	miR-34a active P53 and inactive BCl2 and induces apoptosis	[69] [70]
Yang	2022	CRC	LRPPRC via miR-34a and P53 and 34a/LRPPRC/MDR1 pathway promoted drug resistance	[71]
Singh	2022	CC	miR-34a down regulate Cdt2/DTL and suppress cell transformation and proliferation	[72]
Qi	2022	Liver Cancer	miR-34a active p53 and inhibit MDM2 and Lactate dehydrogenase to prevent cell proliferation	[73]
Tang	2022	TC	Crocin upregulated PTPN4, increased caspase-3 and by miR-34a overexpression, promoted apoptosis	[74]
Ren	2022	BC	miR-34a inhibit KCNQ1OT1 and active Notch3 to prevent proliferation, migration and invasion	[75]
Hong	2023	Hepatocellular, lung, bladder, renal, and ovarian cancers BC, sarcoma,	miR-34a suppresses tumor immune evasion, such as PD-L1 and CTLA-4	[76]
Bouznad	2023	CRC	miR-34a inhibit the IRE1A/XBP-1, EMT, migration, invasion, and chemo-resistance	[77]
Gu	2023	hepatocellular carcinoma	miR-34a inhibited the expression of Beclin-1 and increased sensitivity to drug	[78]
Liu	2023	CRC	miR-34a suppressed migration and invasion by activating P53 and KEAP1/NRF2/ARE pathway	[79]
Deng	2023	BC	miR-34a activated P53 and downregulated PD-L1 and inhibited the cell growth	[80]
Abate	2023	BC	miR-34a activated P53 and inactivated AMPK-SIRT1 axis, ATG4B, beclin-1, and LC3B II/I and induced growth inhibition and apoptosis	[81]

#### 3.1.1 Cell proliferation and growth

miR-34a downregulats in many tumors, such as urinary system cancer (UC). In bladder cancer cell lines 5637 and T24, miR-34a by inhibiting CCND1 and CCNE2 leads to stopped of cell proliferation and cell cycle in the G0/G1 phase. miR-34a also inhibits EMT via targeting ZEB1, Snail, and Twist which are transcription factors for EMT. It upregulates PTEN and inhibits cancer migration and invasion [54]. Another study clarified that cell proliferation is affected by miR-34a through many cell cycle-regulating proteins, such as cyclins and kinases dependent on cyclin (CDK1, CCND1, CDK4, CDK6, cyclin E2, CDC25C, etc.) [55].

#### 3.1.2 Cell invasion and metastasis

miR-34a can downregulate the Wnt signaling pathway to prevent migration and invasion processes. miR-34a has an inhibitor effect on the Wnt/β-catenin signaling pathway, including Wnt1, β-catenin that they are key molecules for cancer invasion [56]. Upnregulated of miR-34a, leads to decreased Sirtuin1 (SIRT1) expression, as an oncogene that promotes expression of Wnt/β-catenin, and reduces the Wnt/β-catenin signaling pathway. All these changes inhibit tumor metastasis [57]. Another pathway related to cancer growth is the GDNF family receptor alpha 1 protein (GFRA1). It is a receptor on the cell surface of the glial cell that is involved in tumorigenesis by regulating migration and invasion. GFRA1, probably upregulation of AKT and Extra-cellular Signal Regulated Kinase (ERK) pathways in cancer cells [58].

#### 3.1.3 Cell apoptosis

miR-34a regulates the transcription factor p53. In Apoptosis, miR-34a directly targets proteins, including BCL2 and SIRT1, and represses their translation. Thus, inhibition of SIRT1 causes an increase in p53 acetylation, resulting in p53 transcriptional activation, followed by cell death and damage. Also miR-34a, by activating apoptotic proteins Bax-Bak, reduces the expression of BCL2, and leads to cell death [55]. miR-34a mediates cell signaling pathways dependent on cancer cell apoptosis including: EGFR, PDGFR-α/β, MET, SIRT1, NOTCH1, BCL2, and HMGB1. Deactivated EGFR and NOTCH1 by miR-34a inhibits PI3K-AKT pathways and turns on apoptosis. miR-34a/c overexpression by downregulating PDGFR-α/β increased the response of TNF-related apoptosis [59].

### 3.2 miR-34a-5p

**Fig. 4:**
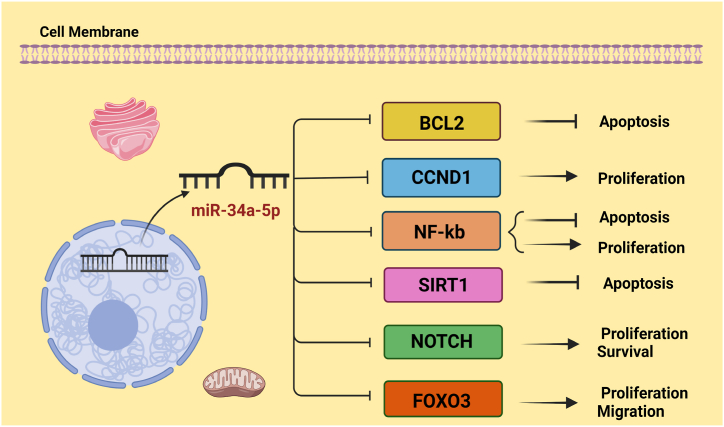
miR-34a-5p has tumor suppressor effects and targets the gene such as BCL2, CCND1, SIRT1, NOTCH, FOXO3, and NF-κβ.

miR-34a-5p has antitumor effects too. Expression of miR-34a-5p has decreased in about 25 % of several cancers [82]. Significant reduction of miR-34a-5p expression in cancer cell lines is associated with cancer treatment resistance. miR-34a-5p targets many signaling molecules such as BCL2, CCND1, and SIRT1 that have a key role in the biological activities of cancer cells (Fig. 4) [83].

#### 3.2.1 Cell proliferation and growth

miR-34a-5p suppresses Head and Neck Squamous Cell Carcinoma (HNSCC) by targeting flotillin 2 (FLOT-2) which is associated with cancer progression. When levels of FLOT-2 expression increased, cell proliferation, EMT induction, and cell cycle progression of HNSCC increased. In HNSCC, miR-34a-5p disrupts cell cycle progression by modulating the MEK/ERK1/2 pathway and suppressing FLOT-2 expression. FLOT-2 Promotes the proliferation and EMT of cancer cells by activating the MEK/ERK1/2 pathway. ERK1/2 by phosphorylating MAPK signaling components, such as factor receptor (FGFR), activated PI3K/AKT, and deactivated P53 promoted cell cycle and angiogenesis [84]. Also, miR-34a-5p suppresses the signaling pathways that are key regulators in metabolic function related to the growth of cancer including Hippo-YAP1/TAZ, TRIM44, and FLSs-RA. Overexpression of TRIM44 and downregulation of Hippo-YAP1/TAZ can reduce E-cadherin and inhibit the malignant behavior of cancer cells, such as proliferation [85,86]. FLSs-RA, by inducing glucose consumption rate and metabolic shift towards increased anaerobic glycolysis, leads to induce mitochondrial phosphorylation in cancer cells [87].

Another important pathway that is mediated by miR-34a-5p is TGF-β1/Smad4. TGF-β1 motivates cell proliferation, and differentiation. Smad proteins are transcriptional factors of TGF-β1 expression. miR-34a-5p in order to cancer proliferation regulation, inhibits TGF-β1/Smad4 signaling pathway [88].

#### 3.2.2 Cell invasion and metastasis

miR-34a-5p targets DAAM1 that obviously upregulated in cancer cell lines. DAAM1 overexpression decreased p21 and increased ERK and AKT that lead to cell migration and invasion [89].

miR-34a-5p have a key function in the CXCL10/CXCL11/CXCR3 axis. In BC cells, miR-34a-5p inhibits CXCL10 and suppresses the invasion and metastasis. Overexpression of miR-34a-5p decreases M1 macrophage synthesis and CXCL10, CXCL11, CD4^+^, and CD8^+^ on the surface of T cells. Also, miR-34a-5p inhibits Lymphoid enhancer-binding factor 1 (LEF1), SIRT1, and NOTCH pathway, and increases acetylated p53 therefore miR-34a-5p inhibits the colon's self-renewal of cancer stem cells. LEF1 is an activator for the Wnt/β-catenin signaling pathway and EMT for the cancer cell invasion [84].

#### 3.2.3 Cell apoptosis

SNAI1, a transcription factor in cancer cells, mediates inhibited apoptosis. It mediated radio resistance and chemo resistance by decreasing in p53 level. Decrees the expression of SNAI1 with up-regulation of miR34a-5p by Apigenin, a natural material of flavone categories, induces p53 and NF-κβ activation and cell death in lung cancer [90]. NF-κβ and p53 independently transcribe miR-34a-5p. The expression of miR-34a-5p and SIRT-1 is associated with each other, and SIRT1 can control the production of anti-oxidative, anti-inflammatory, and vasodilator factors. The increase of SIRT1 inactives P53 and NF-κβ, and reduces inflammation and apoptosis [91,92].

To control cell death miR-34a-5p also regulated putative kinase 1 (PINK1). As a mitophagy initiation factor, PINK1 played an important role in response to stimulus in damaged mitochondria, interacted with Beclin1, Impaired its Pro-Apoptotic Cleavage, and protected against staurosporine-induced Apoptosis. miR-34a-5p promotes apoptosis and acts as a tumor suppressor by downregulating PINK1, which also is known as FOXO3 [93,94].

## 4 Discussion and moving forward

miR-16 and miR-34 families are examples of miRNAs with tumor suppressor function by controlling molecular pathways. Moreover, many studies reported the abnormal downregulation of these miRNAs in malignant tissues. They are modulate cancer proliferation and growth by downregulation of FLOT-2, MEK/ERK1/2, FGFR, PI3K/AKT, TRIM44, FLSs-RA, TGF-β1/Smad4, CCND1, CCNE2, CDK1, CDK4, CDK6, CDC25C, CHEK1,ANLN and VEGFA and upregulation of PTEN. They also decrease the expression of Wnt/β-catenin, SIRT1, CXCL10/CXCL11/CXCR3 axis, CD4^+^, CD8^+^, LEF1, MMp7, and smad3 to inhibition cell invasion and metastasis. The other way that these miRNAs are used to stop and curb cancer is the promotion of apoptosis rate. They modulate the expression of SNAI1, P53, NF-κβ, SIRT1, PINK1, EGFR, PDGFR-α/β, MET, NOTCH1, BCL2, HMGB1, PI3K-AKT, beclin1, CARM1, TAG2, RhoA-p38 MAPK, Bim, Apaf-1, Caspase-9, and Bax/Bcl-2 for increase cell death. Knowing about these mechanisms is effective for improving drugs and treatment methods.

Despite the key effects that these miRNAs have in slowing the progression of cancer, it should be noted that studies still face limitations that highlight the need for more research. Many of the mentioned signaling pathways are influenced by factors such as other miRNAs, and exosome of cells that are present in the tumor microenvironment, which is cleared the point that changing the expression of the miRNAs investigated in this study alone is not enough to control these pathways. A study performed by Peiwen Fu et al. [95] found that extracellular vesicles (EVs) also called exosomes from Human umbilical cord mesenchymal stem cells (hUCMSCs) contained various miRNAs such as miR-193a-3p. They used a fluorescent label in GQDs/Cy5-miR to characterize the effects of this miR in treating tumoral mice. Cy5 is a chemical modification of miRNA attached to GQDs. This attachment was made through “π–π” stacking in the “off” state, and the function of EVs followed by the sonication. Endocytosis was the mechanism of miRNA delivery into cells. miR-193a-3p activated CCND1, and quenched fluorescence and showed tumor suppressor function. In another study, the effects of fucoidan-treated MSC-derived exosomes (F-MSCs-Exo) in osteoarthritis were evaluated under in vitro and in vivo conditions. The results showed that F-MSCs-Exo compared to MSCsExo, are more effective in reducing inflammation and destruction of the extracellular matrix. In this study, miR-146b-5p had a beneficial effect on the F-MSCs-Exo protective cartilage process by inhibiting TRAF6. However, it was stated that due to the unpredictability of the therapeutic effects of exosomes, their extensive clinical application faces limitations [96]. In a contrasting role with the mentioned study, EVs including drug efflux pumps, oncoproteins, antiapoptotic proteins, or microRNAs can lead to cancer drug resistance. For example, EV contains 221 miR, and miR-222 led to drug resistance of breast cancer cells to tamoxifen. As well as, EV- (miR-22-3p, miR-185-5p, miR-503-5p, miR-652-3p, and miR-1280) transfer from drug-resistant breast adenocarcinoma into drug-sensitive cells lead to drug resistance [97]. Therefore, paying attention to the role of exosomes in the process of cancer progression, on the one hand, and other miRNAs in the tumor microenvironment, on the other hand, seems essential in understanding tumor cell mechanisms. In this regard, it has been determined that the expression of miR-3689a-3p significantly reduced the resistance of breast cancer cells to sorafenib by abrogating SOD1-mediated mitochondrial oxidative stress, which ultimately increased HCC cell death [68]. Also. recent studies, investigated the role of miRNAs such as miRNAs −100, miRNAs −99a, and miRNAs −21 on targeting the mammalian target of the rapamycin (mTOR) pathway, a vital pathway for main cellular functions, including cell growth, proliferation, synthesis, and death, which influence on radiosensitivity of cancer cells [68].

In addition, these studies have often been conducted in invitro conditions with small sample sizes, and the role of the tumor microenvironment in the expression of miRNAs and its target pathways has been neglected. Therefore, it seems necessary to investigate the changes in the tumor environment in the human body, however, it is difficult to manage studies in invivo conditions in the human body. The existence of various factors such as underlying diseases, gene mutations, the situation of the patient's immune system, living and economic conditions, and even the situation of nutrition can severely limit studies. Therefore, most of the studies are still limited to the laboratory environment and the animal body. There are differences between the conditions of the animal and human body which are other limitations for declaring a definite opinion about the effect of drugs on the change of the expression of miRNAs and its signaling pathways, which affects the success of the treatment and increases the risk of its failure. We strongly recommend that future studies be performed in three-dimensional co-culture conditions with other cells present in the tumor environment and by using instruments such as microfluidics, which provide the possibility to simulate the dynamic microenvironment. In addition, it is suggested that future research focus on the effect of drugs based nanoparticles and compare their performance on each of the signaling pathways to find a useful drug. based on many studies including the study of Sharon Wei Ling Lee et al. [98] encapsulation of miRNAs into NPs can overcome to delivery challenges of miRNAs including efficient and safe delivery.

## 5 Conclusion

Among the miRNAs, miR-16 and miR-34 families are tumor suppressors. They have functional roles in gene expression. Also, they can control necessary cellular functions such as proliferation, differentiation, and cell death and may provide avenues for cancer therapy. These miRNAs provide valuable information about the complex factors that can be overexpressed and impact cancer progression. Also, identifying these miRNAs and their mechanism of action can help identify the progress of cancer, choose, and improve cancer treatment methods. Hence, research on this issue should be pursued.

## CRediT authorship contribution statement

**Zahra Sadeghi:** Writing – original draft. **Mehrnoush Malekzadeh:** Writing – review & editing. **Mohammadreza Sharifi:** Investigation. **Batool Hashemibeni:** Conceptualization.

## Ethics approval

Not applicable. This article does not contain any studies with human participants or animals.

Performed by any of the authors.

## Consent for publication

Not applicable.

## Funding

Not applicable.

## Declaration of competing interest

The authors declare that they have no known competing financial interests or personal relationships that could have appeared to influence the work reported in this paper.

## Figures and Tables

**Table:** 

PdPK1	3-phosphoinositide-dependent protein kinase-1
BMI1	B lymphoma Mo-MLV insertion region 1
BCL2	B-cell lymphoma 2
CLL	Chronic lymphocytic leukemia
CCND1	Cyclin D1
EMT	Epithelial-mesenchymal transition
ECM	Extracellular matrix
HNSCC	Head and Neck Squamous Cell Carcinoma
LEF-1	Lymphatic enhancer factor 1
MSCs	Mesenchymal stem cells
miRNAs	MicroRNAs
PRC1	Polycomb Repressor Complex 1
SCLC	Small-Cell Lung Cancer
TGF-βR1	TGF-β receptor 1
TNBC	Triple-Negative B cells
